# Elastic Properties of BCN Alloys, Graphene, and *h*‑BN Monolayers Containing Point Defects

**DOI:** 10.1021/acsomega.5c10085

**Published:** 2026-02-16

**Authors:** Prosun Santra, Mahdi Ghorbani-Asl, Sadegh Ghaderzadeh, Elena Besley, Arkady V. Krasheninnikov

**Affiliations:** † Institute of Ion Beam Physics and Materials Research, 28414 Helmholtz-Zentrum Dresden-Rossendorf, 01328 Dresden, Germany; ‡ School of Chemistry, 6123 University of Nottingham, Nottingham NG7 2RD, U.K.

## Abstract

Point defects can strongly affect the mechanical properties of two-dimensional (2D) materials, causing an overall detrimental effect on the strength, stiffness, and elasticity. However, the opposite has also been reported in the literature, which indicates that our understanding of the role of defects at the atomic level remains incomplete. This computational study provides a systematic assessment, based on first-principles calculations, of the mechanical properties of the archetypal 2D materials (*h*-BN and graphene monolayers) containing substitutional impurities and vacancies, which is further extended to 2D BCN alloys representing the case of high concentration of substitutional impurities in *h*-BN and graphene. In general, the stiffness of these materials, as described by Young’s modulus, decreases in the presence of point defects. The Young’s modulus of *h*-BN decreases rapidly with increasing concentration of C atoms in the N positions, while the drop is smaller for C impurities in the B positions. Notably, a defect configuration, in which carbon atoms replace the neighboring N and B atoms as a pair, results in the values of the Young’s modulus in the range between that of pristine graphene and *h*-BN. In *h*-BN, B vacancies give rise to a greater decrease in stiffness than N vacancies, as explained by the analysis of the local defect-mediated strain fields formed near the point defects. The effects of graphene weakening through the introduction of substitutional defects and vacancies are similar to those observed in *h*-BN. This mechanical behavior persists in materials with few atomic percent of point defect concentration and agrees with most experimental results found in the literature. As the mechanical properties of 2D BCN alloys can be manipulated by a preferential substitution of B and N atoms with C atoms, our predictions may guide future efforts in defect-mediated engineering of the mechanical properties of 2D materials.

## Graphical abstract

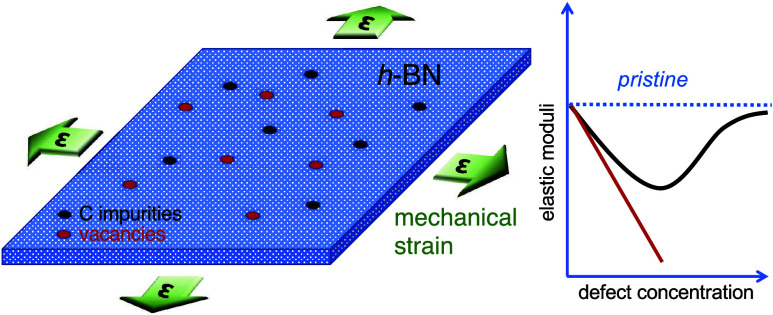

## Introduction

Controlled introduction of defects and impurities into materials is a common route to tailoring their mechanical properties.^1^ For example, adding phosphorus to steel has been demonstrated^2^ to increase its strength and hardness. Other impurities like carbon and nickel,^1,3^ along with extended defects such as dislocation networks^4^ and grain boundaries,^5–7^ have been routinely used for specific purposes, e.g., to control the ductility and hardness. Putting aside the reinforcement of soft materials, such as making composites^8–11^ and alloying,^12–14^ the general expectation is that structural imperfections should decrease the Young’s modulus, as the defects typically give rise to the weakening of chemical bonds and even the formation of voids.

Point defects and impurities can be introduced into two-dimensional (2D) materials during the growth^15,16^ or by postsynthesis techniques like ion irradiation,^15,17–19^ atom deposition,^20^ or by chemical treatment.^15,21^ The presence of point defects, even at low concentrations, has been found to drastically affect the electronic, optical, magnetic, and catalytic properties of 2D systems.^15,22–24^ This effect can be advantageous depending on the type of defects and their concentration. For example, defects can be used to realize single-photon emitters in 2D materials,^25–27^ add new functionalities like magnetism,^28,29^ enhance catalytic activity,^23^ or modify electronic properties.^30,31^ They can also alter the mechanical characteristics of 2D materials to a greater degree than those in bulk due to the reduced dimensionality.

Graphene has garnered significant attention in that context. The predicted values of the Young’s modulus^32,33^ and tensile strain^34^ of graphene are very high, but the experimentally measured values were not always close to the theoretical limit.^35,36^ The apparent discrepancy is not fully understood. Ion irradiation of graphene combined with the nanoindentation measurements using atomic force microscopy has been employed to study the effect of defects on mechanical properties of graphene.^37^ For a vacancy concentration of 0.2%, the experiments^37^ provided some puzzling results. The elastic modulus of graphene was reported to increase upon introduction of defects, nearly doubling its value as compared to the pristine graphene layer, which was attributed to the suppression of flexural phonon modes at longer wavelengths. The increase in the Young’s modulus in the presence of vacancies in graphene has been also found in later experiments.^38^ However, the standard fracture continuum models predicted the fracture strain to decrease with defect concentration. We note that in the study,^37^ the Young’s modulus was not obtained directly from a stress-strain curve but assessed using a model derived for a macroscopic membrane.

These results were not reproduced in other studies. In contrast, it was reported^39^ that the 2D elastic modulus and strength of graphene remain largely unaffected by the introduction of vacancies, even at 5 nm separation. It was also demonstrated that adatoms, not vacancies, improve the mechanical properties of graphene, such as the elastic modulus and tensile strain.^39^ In a very recent study,^40^ the changes in the 2D elastic modulus of the pristine and defective graphene have been studied by a combination of scanning transmission electron microscopy and atomic force microscopy nanoindentation. The experiments^40^ were carried out in ultrahigh vacuum, and the defects were created in situ by low-energy ion irradiation. A rapid decrease in the 2D elastic modulus from 286 to 158 N/m was detected at a vacancy concentration as low as 1.0 × 10^13^ cm^–2^ (atomic concentration of 0.26%) and investigated using classical molecular dynamics (MD) simulations. These simulations related the softening of graphene to structure corrugations caused by the local strain formed near the vacancy sites with two or more missing atoms, while the influence of single vacancies was shown to be negligible.

Other theoretical works have extended these studies from graphene to *h*-BN, another truly 2D material. Extensive classical MD^41–47^ and tight-binding^48^ simulations of defective graphene and *h*-BN^49,50^ suggested that point defects should have a detrimental effect on the mechanical properties by reducing the tensile strength and elastic modulus of both 2D materials. Classical MD results should be treated with caution, as typically the accuracy of classical potentials is not tested systematically for defective materials. Density functional theory (DFT) calculations^51^ also indicated that the value of the Young’s modulus of graphene goes down with the increase in vacancy concentration. However, the supercell size in these DFT calculations was relatively small and may have caused an unphysical interaction between the images of the defects through the elastic strain fields.^52^ Furthermore, a recent DFT study^53^ demonstrated that the formation energy of point defects can either increase or decrease with the externally applied biaxial strain, depending on the atomic radius of impurity atoms, which can be larger or smaller than the host atom. This can cause additional local defect-induced compressive or tensile strains located at impurity sites, which affect the external strain. It was suggested that the change in the defect formation energy can be related to the change in the value of the elastic modulus upon the introduction of impurities.

Another interesting class of 2D materials whose mechanical characteristics have been investigated experimentally includes BCN alloys.^54^ Mechanical properties of a mixed BCN layer strongly depend on the carbon content. Reducing the content of boron and nitrogen to a very small amount (atomic concentration of 2.5%) makes it possible to synthesize a BCN film with similar properties to pure carbon film but with better wear resistance.^54^ The mechanical characteristics of layered BCN alloys, which can be referred to as graphene with a high concentration of B and N impurities or *h*-BN with carbon substitutions, have been shown to lie in the range between those of pure graphene and *h*-BN.^55^ However, the effect of preferential defect substitution has not been addressed. Despite several experimental and theoretical studies of the mechanical properties of monolayer graphene, *h*-BN, and BCN alloys, an understanding at the atomic level of the role of defects remains incomplete. In this work, we undertake a systematic assessment of the effect of point defects in these 2D materials by carrying out first-principles calculations and complete the study by considering BCN alloys, which represent the case of a high concentration of substitutional impurities.

## Results and Discussion

### Mechanical Properties of BCN Alloys with Different Stoichiometries

The Young’s modulus, *Y*, and 2D elastic modulus, *Y*_2D_, have been calculated for graphene and *h*-BN with substitutional impurities, which can also be viewed as BCN alloys, for different defect concentrations, e.g., carbon content (see the Methods section for details). The Young’s modulus has been evaluated by applying the uniaxial strain in armchair or zigzag directions. When calculating *Y*_2D_, the strain was applied in both directions. For *h*-BN, two types of substitutional impurities, C_B_ and C_N_, have been considered, where a carbon atom replaces one of the host boron or nitrogen atoms.^64,65^ In graphene, both B and N atoms in substitutional positions have been studied, B_C_ and N_C_, respectively. We note that the maximum concentration of C_B_ defects is 50%, at higher carbon concentrations, boron atoms are not present, so that the system can be referred to as graphene with N impurities, and the notation N_C_ is used. The connections between the C_N_ and B_C_ defects are similar. A random distribution of defects also included an atomic configuration, frequently observed in *h*–BN,^66^ in which carbon atoms replace the neighboring N and B atoms (pairwise substitution). We did not study more complicated substitutional multicarbon defects in *h*-BN,^65,67^ as the complexity of the system, that is, the number of possible configurations, increases rapidly, but we do not expect any qualitative changes in the behavior of the system under strain. We did not account for topological (Stone–Wales) defects with carbon substitution^68^ either, as they are not common in graphene and *h*-BN, as evident from numerous experimental TEM and STM studies, see ref 69 for an overview.

For each distribution and concentration of the defects, the supercell size of the systems without strain was carefully optimized. The optimization of the supercell size is very important for a quantitatively accurate evaluation of the mechanical responses of defective 2D materials to external strain, especially at higher concentrations of defects (above 2%). The change in the average size of the unit cell as a function of carbon concentration is shown in Figure 1. The left-hand side of the figure at 0% carbon concentration corresponds to pristine *h*-BN, and the right-hand side at 100% carbon concentration gives the results for graphene without impurities. The dependence can be understood in terms of the atomic radii of B, C, and N atoms, which decrease from B to N. The changes in the average size of the unit cell, shown in Figure 1, reduce the effect of the local strain and decrease the value of the elastic modulus, as discussed below. Lattice parameters are also listed in Table 1, along with the average cohesive energies. We note that for the pairwise substitution, the mixing energy (the energy difference between the BCN system and pure graphene and *h*-BN) is positive in agreement with the results of previous calculations,^70^ indicating that segregation of BNC to graphene and *h*-BN is energetically favorable. The same is true for single-atom substitution, although the energy penalty depends in this case on the environment (N-rich/B-rich conditions), as demonstrated earlier.^64^

**Table 1:** Cohesive Energies of the Mixed BCN Systems with Various Carbon Concentrations^a^

system	carbon concentration (%)	average cohesive energy (eV/atom)	average unit cell parameter (Å)
C_B_	0	–7.06	2.511
	2	–7.01	2.505
	8	–6.84	2.488
	15	–6.63	2.471
	25	–6.43	2.447
	35	–6.2	2.407
	50	–6.38	2.390
	65	–7.03	2.431
	75	–7.23	2.436
	92	–7.75	2.453
	98	–7.92	2.459
	100	–7.97	2.465
C_N_	0	–7.06	2.511
	2	–7.03	2.517
	6	–6.96	2.534
	15	–6.82	2.574
	25	–6.72	2.598
	40	–6.58	2.651
	50	–5.72	2.678
	65	–6.44	2.621
	75	–6.94	2.574
	92	–6.08	2.499
	98	–6.09	2.471
	100	–7.97	2.465
pairwise	25	–7.09	2.510
	50	–7.18	2.499
	75	–7.42	2.488

a Cohesive energies are calculated with respect to isolated atoms.

**Figure 1:**
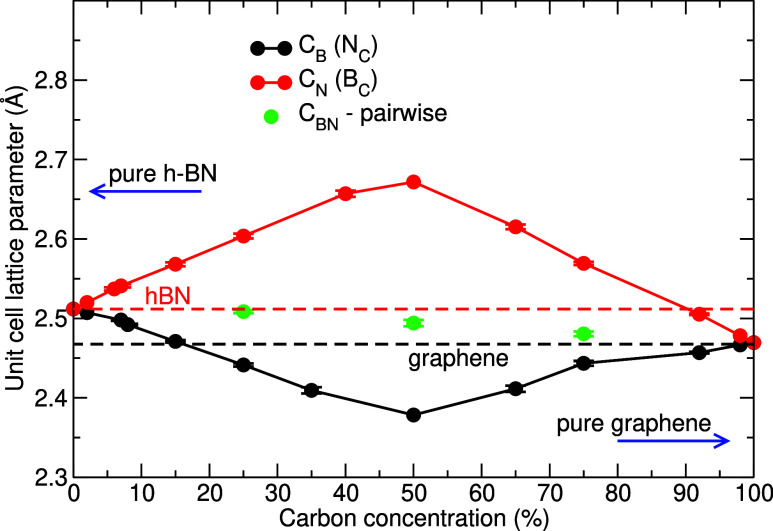
Average lattice parameter for the unit cell corresponding to a mixed 2D BCN layer as a function of carbon concentration; 0% carbon concentration corresponds to pristine *h*-BN and 100% carbon concentration corresponds to pristine graphene.

Figure 2 presents the key results for the changes in the Young’s modulus of materials containing C_B_ (N_C_) and C_N_ (B_C_) defects depending on the concentration of carbon. The calculated values for the Young’s modulus of pristine *h*-BN and graphene agree well with previous experimental and theoretical results.^71^ In these materials, Young’s modulus has been found to be slightly lower along the zigzag direction than in the armchair direction, in both pristine and defective forms, regardless of the type of defects. The horizontal dashed lines in Figure 2 indicate the Young’s modulus of pristine graphene (black) and *h*-BN (red) along the armchair (Figure 2a) and zigzag (Figure 2b) directions.

**Figure 2:**
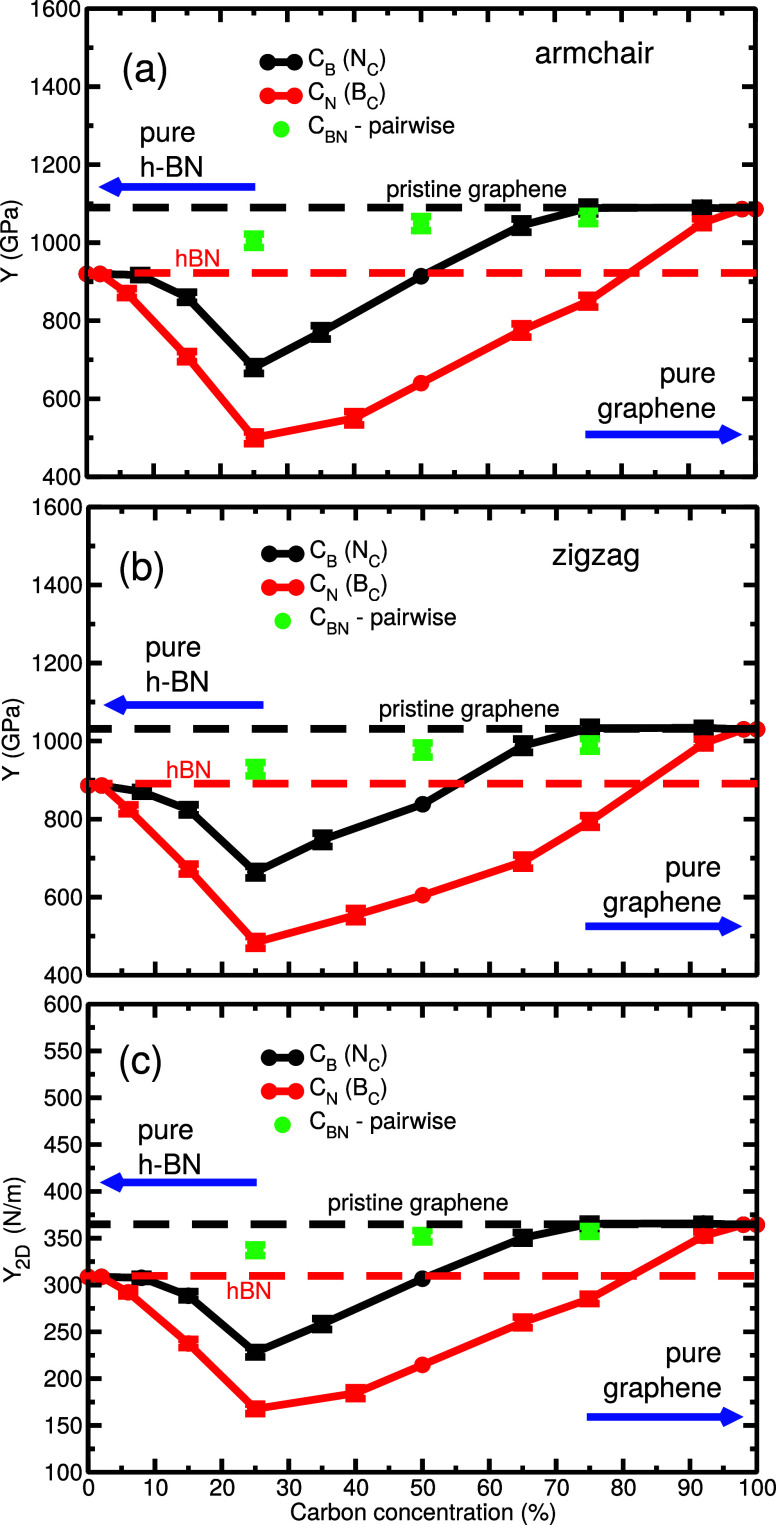
Young’s modulus, *Y*, of the mixed *h*-BN/graphene system containing C_B_ and C_N_ defects as a function of carbon concentration: (a) uniaxial strain is applied in the armchair direction and (b) in a zigzag direction. The 2D elastic modulus, *Y*_2D_, is presented in panel (c). The left-hand side of the plots corresponds to pure *h*-BN and the right-hand side to graphene. Horizontal lines indicate the values of the Young’s modulus of pristine graphene and *h*-BN. Random pairwise substitutions (C in both B and N positions) at several concentrations (25%, 50%, and 75%) are also considered. Error bars represent the standard deviation.

Introducing a small amount of carbon impurities to pristine *h*-BN results in the value of the Young’s modulus to drop and reach the minimum at 25% of carbon concentration. The Young’s modulus of *h*-BN containing C_B_ defects decreases at a slower rate and has consistently higher values than that of *Y* of *h*-BN with the same concentration of C_N_ defects. In the case of graphene with low concentrations of B/N impurities, Young’s modulus decreases with the reduction of carbon content. However, boron substitution, B_C_, gives rise to a rapid decrease in the value of *Y*, while nitrogen substitution, N_C_, does not introduce any noticeable change in *Y* until the concentration of carbon is reduced by at least 25%. The trends for the armchair (Figure 2a) and zigzag (Figure 2b) directions are the same. In the case of random simultaneous substitution of C_B_ and C_N_ at carbon concentration of 25%, 50%, and 75% (labeled as C_BN_ in Figure 2), the value of the Young’s modulus falls between *Y* of pristine graphene and *h*-BN, which is in agreement with previous results.^55^ The dependence of the 2D elastic modulus, *Y*_2D_, on carbon concentration (Figure 2c) matches well with the results for Young’s modulus.

To understand these quantitative differences in the observed mechanical behavior, we calculated the difference in the bond distribution function, Δρ, as described in the Methods section. As shown previously,^53^ point defects can give rise to a local, defect-mediated strain that can affect the overall elastic energy–strain dependence. Figure 3 presents the bond distribution function for C_B_ and C_N_ defects in *h*-BN at the concentrations of 0.4%, 2%, and 7%, which display various degrees of bond contraction and elongation affecting the local area near the defect.

**Figure 3:**
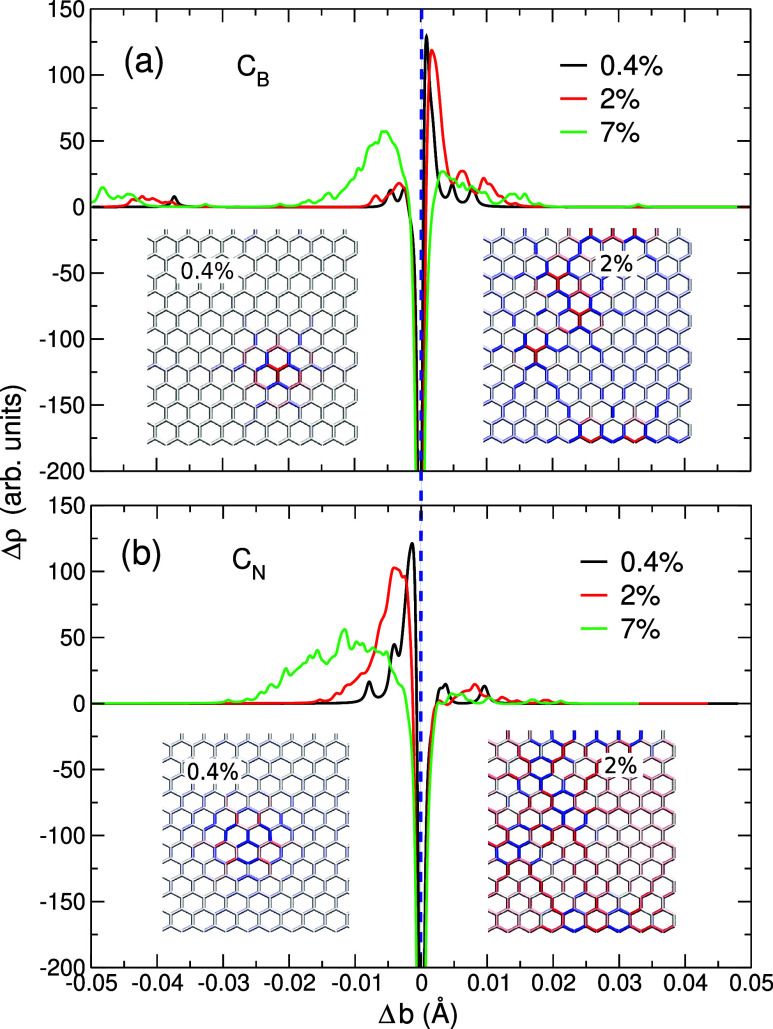
Difference in the bond distribution function, Δ*ρ*, for *h*-BN containing C_B_ defect (a) and C_N_ defect (b) at the carbon concentration of 0.4%, 2%, and 7%. The vertical dashed line indicates the bond length of pristine *h*-BN. Insets are the strain maps at the carbon concentration of 0.4% and 2% for C_B_ (a) and C_N_ (b); blue indicates bond elongation relative to the bond length in pristine *h*-BN, and red indicates bond contraction.

In the case of C_B_ defects in *h*-BN (Figure 3a) with the concentration of 0.4% and 2%, there is an overall elongation in the bond length as the difference in Δρ is significantly greater for longer bonds than for shorter bonds. As discussed previously,^53^ such a mechanical response to the externally applied strain is associated with the size of the atomic radius of carbon, which is smaller than that of the boron atom. In this case, the bonds nearest to the carbon impurity are shorter than those in pristine *h*-BN and cause a local tensile strain. The formed strain field is anisotropic and localized in the vicinity of the defects, with a range of about 8 Å. For higher concentrations of the impurities, the defect-induced compressive and tensile strain fields overlap, and the overall effect is weakened.

In contrast, for C_N_ defects (Figure 3b), Δρ is larger for shorter bonds, which indicates the overall compression of the material. Such behavior is also expected as the atomic radius of carbon is larger than that of nitrogen, leading to the elongation of the bonds near the C_N_ impurity. This gives rise to the overall compressive strain (overall bond contraction) in the area close to the defect. The external tensile strain then compensates for the local defect-induced compressive strain, thus lowering the total energy of the system and the values of *Y* for this kind of substitution. However, at small defect concentrations, the local strengthening of the bonds does not affect noticeably the elastic moduli. At higher concentration, the strain fields compensate each other, and also the average size of the primitive cell changes, as shown in Figure 3. The changes in the average size of the primitive cell in Figure 1 overall diminish the effects of local strain and give rise to the decrease in elastic moduli.

The effects of graphene weakening through the introduction of substitutional defects (Figure 4) are similar to those observed in *h*-BN, as they are also related to the difference in the atomic radii of the host and impurity atom and the associated local strain fields. The introduction of N_C_ impurities (Figure 4a) at small concentrations gives rise to the overall bond elongation near the defect; however, the effect diminishes at larger defect concentrations (e.g., 10%), due to the overlap of the local strain fields. For B_C_ defects (Figure 4b), the impurities give rise to the overall compression in the material as Δρ is much higher for shorter bonds. The trend remains consistent with the increasing impurity concentration, resulting in monotonic behavior and a faster decrease in *Y*. We note that the difference in the atomic radii between B and C atoms (85 vs 76 pm) is larger than that between C and N (76 vs 71 pm), which can explain the stronger effect of B impurities on graphene elastic moduli.

**Figure 4:**
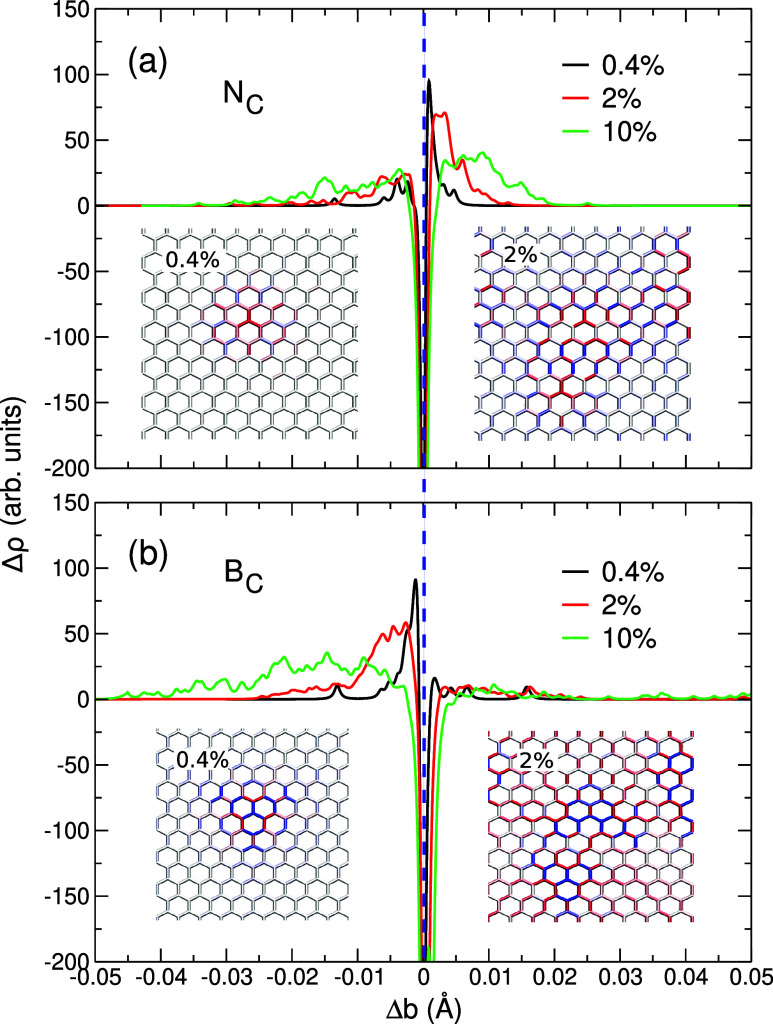
Difference in the bond distribution function, Δρ, for graphene containing N_C_ defect (a) and B_C_ defect (b) at the defect concentrations of 0.4%, 2%, and 10%. The vertical dashed line indicates the bond length of pristine graphene. Insets are the strain maps at the defect concentrations of 0.4% and 2% for N_C_ (a) and B_C_ (b); blue indicates bond elongation relative to the bond length in pristine graphene, and red indicates bond contraction.

### Young’s Modulus of *h*-BN and Graphene Containing Vacancies

We next investigate the dependence of Young’s modulus on vacancy concentration in *h*-BN and graphene. Single vacancy is a common point defect in *h*-BN, which can be preferentially (B or N vacancies) created using electron or ion beam irradiation^26,72–74^ with the specific energy of the impinging particles. As the formation energy of the vacancy depends on the electron chemical potential,^65,75,76^ electrostatic charging of *h*-BN can be also used to create vacancies of a specific type. Figure 5 presents the dependence of the Young’s modulus of *h*-BN in the armchair and zigzag directions on the concentration of vacancies. In all cases, an increase in the concentration of vacancies results in a decrease in *Y*, as reported previously (Table 2). However, the gradient of the drop varies slightly for *h*-BN with B and N vacancies, and the Young’s modulus decreases more slowly in the presence of N vacancies than B vacancies.

**Table 2:** Young’s Modulus of the Pristine, *Y*_0_, and Defective, *Y*, Graphene and *h*–BN Calculated Using Different Methods^a^

system	defect type	concentrations	method	*Y*_0_ (TPa)	*Y*/*Y*_0_ at 1%	*Y*/*Y*_0_ at 5%	references
*h*-BN	V_N_ + V_B_	0%–5%	AP-MD	0.675	0.925	0.55	^56^
	V_N_ + V_B_	0%–14%	AP-MD	0.7	0.942	0.785	^57^
	V_N_ + V_B_	0.1%–1%	AP-MD	0.7	0.571		^58^
	V_N_	0%–4%	DFT	0.91	0.912	0.59	this work
	V_B_	0%–4%	DFT	0.91	0.839	0.47	this work
graphene	V_C_	0.2%–5%	finite-elements	1.2	0.975	0.75	^59,60^
	V_C_	0%–14%	AP-MD	1.03	0.985	0.95	^61^
	V_C_	0.1%–5%	finite-elements	1.2	0.991	0.83	^62^
	V_C_	0%–4%	AP-MD	1.1	0.97	0.88	^43^
	V_C_	0%–6%	AP-MD	1	0.969	0.56	^45^
	V_C_	1%–9%	finite-elements	1	0.993	0.97	^44^
	V_C_	1%–9%	finite-elements	1.3	0.923	0.615	^46^
	V_C_	0.04%–0.2%	tight binding	0.97	0.968		^48^
	V_C_	0%–2%	AP-MD	0.98	0.918		^47^
	V_C_	0%–3%	AP-MD	0.91	0.88		^63^
	V_C_	0%–4%	DFT	1.08	0.96	0.61	this work

a AP-MD stands for analytical potential molecular dynamics.

**Figure 5:**
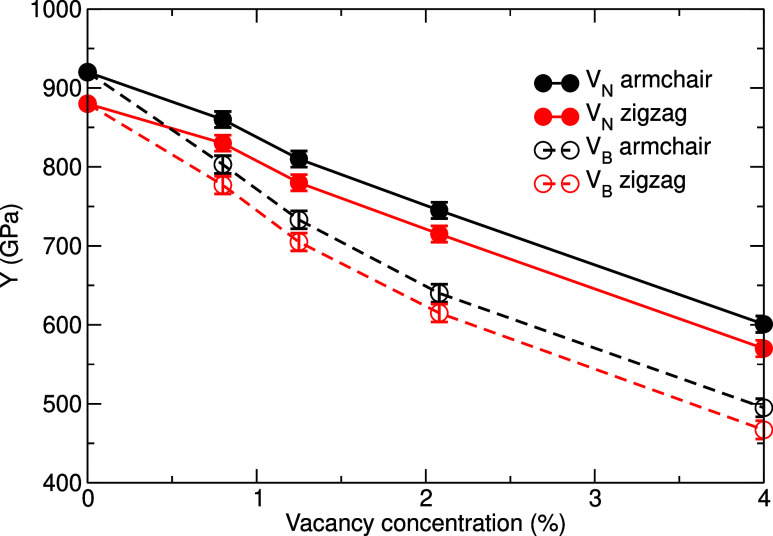
Young’s modulus of *h*-BN with nitrogen, V_N_, and boron, V_B_, vacancies as a function of the vacancy concentration.

The difference in the atomic geometry of V_N_ and V_B_ is evident from the insets in Figure 6. While the removal of the N atom leads to the reconstruction and formation of a new bond between boron atoms, this does not occur in the case of a B vacancy. Thus, an anisotropic tensile strain field is created near V_N_, which is confirmed by the shape of the bond distribution function Δρ shown in Figure 6a. The defect-mediated tensile strain makes the material more strain-resistant, similar to the case of C_B_ impurities in *h*-BN and N_C_ impurities in graphene, but this effect cannot compensate for the missing bond. At the same time, B vacancies give rise to the more localized strain fields, and the overall compressive strain dominates in the material (Figure 6b).

**Figure 6:**
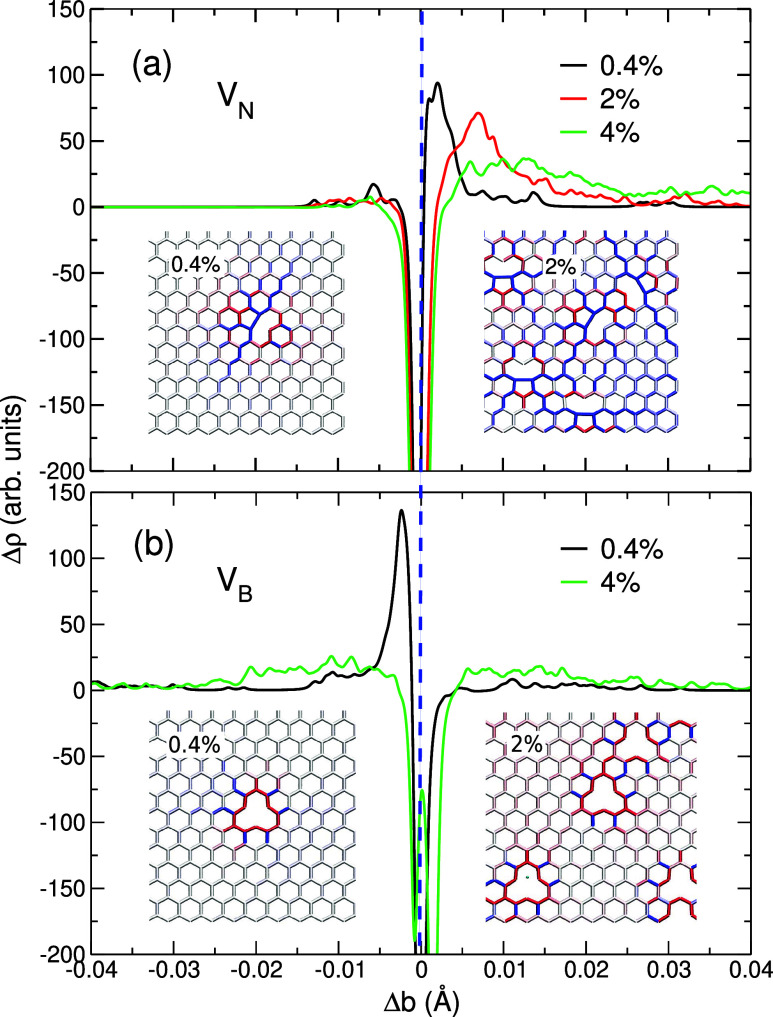
Difference in the bond distribution function, Δρ, for *h*-BN containing N vacancies, V_N_ (a), and B vacancies, V_B_ (b), at the vacancy concentration of 0.4%, 2%, and 4%. The vertical dashed line indicates the bond length of pristine *h*-BN. Insets show the atomic configuration of the vacancies and the associated strain maps at the vacancy concentration of 0.4% and 2% for V_N_ (a) and V_B_ (b); blue indicates bond elongation relative to the bond length in pristine *h*-BN, and red indicates bond contraction.

For completeness, we also studied the dependence of *Y* on the vacancy concentration in graphene, shown in Figure 7, to confirm that it decreases with the vacancy concentration. This generally agrees with the results of numerous previous calculations summarized in Table 2. Up to the concentration of about 1%, the changes are rather weak, but once the concentration of vacancies exceeds 1%, the Young’s modulus decreases noticeably. The Jahn–Teller distortion, typical of the vacancy structure in graphitic systems,^77–79^ is associated with the formation of a new bond and it gives rise to an additional local tensile strain. As graphene is an elastic material, the areas of both tensile and compressive strain are formed locally.^52^ However, in graphene, the bond elongation dominates and creates the overall combined tensile strain, as shown in Figure 8. As discussed above, at higher concentrations of vacancy defects, the induced strain fields overlap and cancel out, leading to a drop in the value of *Y*, in addition to the loss of material through the formation of vacancies.

**Figure 7:**
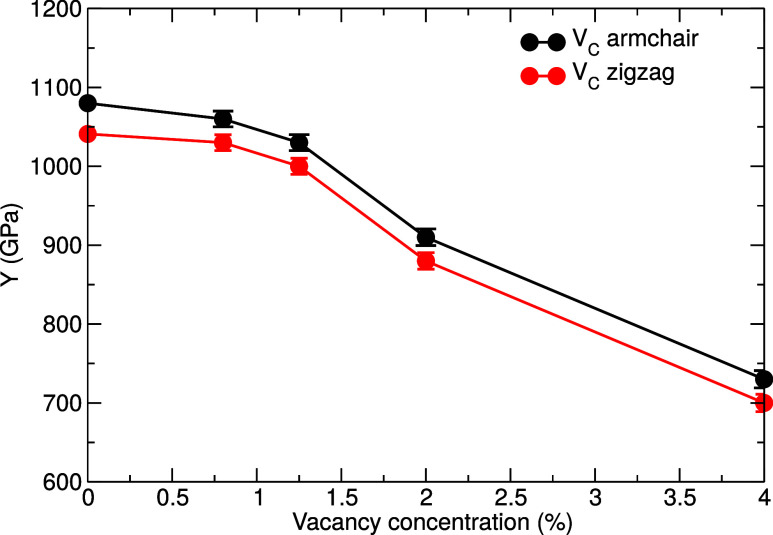
Young’s modulus of graphene containing vacancies as a function of the vacancy concentration.

**Figure 8:**
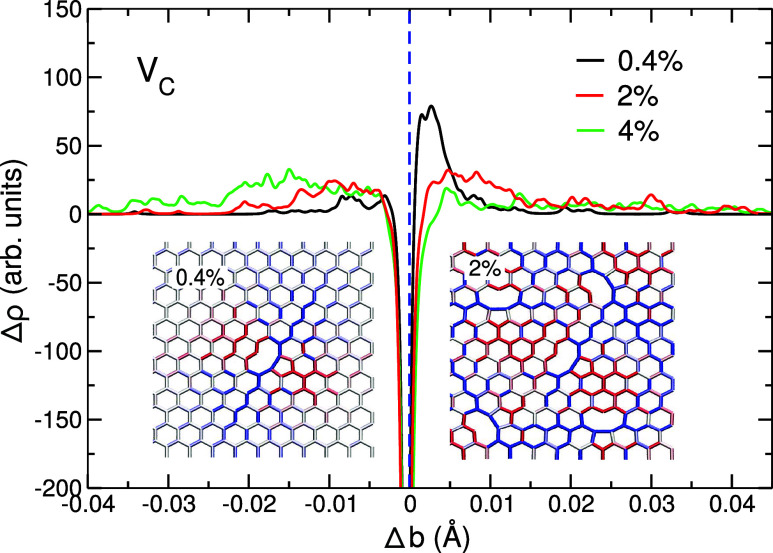
Difference in the bond distribution function, Δρ, for graphene containing vacancies at vacancy concentrations of 0.4%, 2%, and 4%. The vertical dashed line indicates the bond length of pristine graphene. Insets show the atomic configuration of the vacancies and the associated strain maps at the vacancy concentration of 0.4% and 2%; blue indicates bond elongation relative to the bond length in pristine graphene, and red indicates bond contraction.

### Conclusions

In conclusion, first-principles calculations that include optimization of the supercell size indicate that point defects, such as vacancies and substitutional impurities, always result in deterioration of the mechanical properties of archetypal 2D materials, but their effects can be quantitatively different. In 2D BCN alloys, which can be referred to as *h*-BN with a high concentration of C impurities or as graphene with B and N impurities, the dependence of the Young’s modulus and 2D elastic modulus on the carbon concentration is not trivial, and the preferential substitution of B and N atoms with C atoms results in different behaviors. We found that *Y* of *h*-BN decreases rather quickly with the increasing concentration of C atoms in the N location, but the drop is smaller for C impurities in the B location. The stiffness, as described by the Young’s modulus, is lower for the preferential substitution than for pristine material, while it has intermediate values for the substitution of the neighboring B and N atoms as a pair. B vacancies in *h*-BN give rise to a larger decrease in stiffness compared to N vacancies. The differences are explained by the analysis of the defect-mediated strain fields formed near the defects. The observed mechanical behavior is consistent in the range of few atomic percent of the defect concentrations and across different types of point defects.

For defective graphene, static calculations at zero temperature show a decrease in the values of the elastic modulus when defects are present, which is in line with most (but not all) experimental data reported in the literature. Specifically, the increase in the 2D modulus of irradiated graphene reported in ref 37 contradicts our findings, but it can be explained by surface contamination.^40^ We note that the mechanical response of multilayer structures with vacancies and impurities is expected to be very close to the average over the individual responses of each layer, unless a concentration of interstitial-type defects^80,81^ bridging the neighboring layers is high. As for BCN materials, our computational predictions can be verified experimentally through the preferential substitution of the host atoms in graphene and *h*-BN with specific impurities by using chemical treatment or low-energy ion implantation.

## Methods

### Density Functional Theory Calculations

Density functional theory (DFT) calculations have been carried out using generalized gradient approximation (GGA) within the Perdew–Burke–Ernzerhof parametrization^82^ as implemented in the VASP code.^83,84^ The vacuum space of 16 Å in the direction perpendicular to the layer was used for both *h*-BN and graphene to avoid the spurious interlayer interactions. The full geometry and unit cell size optimizations were performed with the force tolerance of 0.01 eV Å^–1^ and a plane-wave cutoff of 400 eV. The Brillouin zone of the unit cell was sampled using a 12 × 12 × 1 *k*-point mesh. In the simulations involving vacancies, V_C_, V_N_, and V_B_, a rectangular supercell composed of 10 × 6 unit cells totaling 240 atoms was used. In the simulations of substitutional defects, C_B_ and C_N_, a rectangular supercell of 4 × 3 including 48 atoms was used. For smaller defect concentrations, i.e., 0% to 7%, 93% to 100%, and specifically 50%, we also calculated the Young’s modulus using rectangular supercells of 10 × 6 with 240 atoms. In the simulations of C_BN_ defects, we also used a rectangular supercell of 10 × 6 with 240 atoms.

The supercell size optimization for *h*-BN and graphene containing different concentrations of defects was carried out, and for each concentration of defects, we considered 10 different configurations. Then we applied a compressive or tensile strain of 0.2% along the armchair and zigzag directions for each configuration. After that the Young’s modulus was calculated numerically. In practice, for each value of strain, the average total energy of the system was first calculated, then the dependence of the energy on strain was approximated by a polynomial to calculate the elastic moduli more accurately. We note that while the size optimization of the 240-atom supercell had no effect on the accuracy of predictions for 2D materials containing isolated defects, it became crucial for defect concentrations above 2%.

The Young’s modulus, *Y*, was calculated as follows

1 Y=F/Aϵ

2 F=[E(+ΔL)−E(−ΔL)]/2ΔL

3 $$\epsilon =\Delta L/L_{0}$$

where *F* is the force exerted on the system due to the external strain ϵ. *E*(+Δ*L*) and *E*(−Δ*L*) are the total energy of the configuration with 0.2% tensile strain and with 0.2% compressive strain, respectively. Here, Δ*L* = |*L* – *L*_0_| is the elongation or compression along the zigzag/armchair direction for 0.2% strain. *A* is the area upon which the force *F* is exerted. For calculations of the Young’s modulus along the armchair/zigzag direction, *A* is expressed as

4 A=Dl

Here *D* is the interlayer distance of *h*-BN = 3.30 Å or of graphene = 3.34 Å and *l* is the lattice parameter along the zigzag/armchair direction.

To examine the changes in the atomic structure after incorporating the impurities and vacancies, we calculated the difference Δρ­(*r*) in the bond distribution function ρ­(*r*) for both defective and pristine systems

5 $$\Delta \rho \left(r\right)=\rho_{\mathrm{def}}\left(r\right)-\rho_{\mathrm{pristine}}\left(r\right)$$

6 $$\rho \left(r\right)=\sum_{i\neq j}\delta \left(r-r_{\mathrm{ij}}\right)$$

where *r*_*ij*_ is the bond length between the nearest neighbor atoms *i* and *j* at zero temperature and δ­(*r*) is the Dirac delta function, which was smeared for a better visualization and understanding of the localization and scattering of the bond lengths.
